# High thresholds encouraging the evolution of cooperation in threshold public-good games

**DOI:** 10.1038/s41598-020-62626-3

**Published:** 2020-04-03

**Authors:** Kris De Jaegher

**Affiliations:** Utrecht University School of Economics, Utrecht University, Utrecht, The Netherlands

**Keywords:** Evolutionary theory, Social evolution

## Abstract

For a well-mixed population, we consider a threshold public good game where group members only obtain benefits from a public good if a sufficiently large number of them cooperates. We investigate the effect of an increase in the threshold on the level of cooperation that evolves. It is shown that for sufficiently large participation costs, the level of cooperation is higher for low and for high thresholds, than it is for intermediate thresholds – where in the latter case cooperation may not evolve at all. The counterintuitive effect where an increase in the threshold from an intermediate to a high one decreases the probability of cooperation, is related to the so-called common-enemy hypothesis of the evolution of cooperation. We further apply our analysis to assess the relative weight of different game types across the parameter space, and show that game types where either a small, or a large fraction of the population evolves as cooperators, receive more weight compared to game types where an intermediate fraction of cooperators evolves.

## Introduction

Cooperative behavior is characterized both by social diversity, namely the fact that some organisms have high levels of cooperation and others do not^1^, and by phenotypic plasticity, where individual organisms cooperate in some contexts but not in others^2^. At the same time, key examples of cooperation between organisms can be modeled as so-called threshold public-good games^3–9^ (also known as participation games^10,11^, discrete public-good games^12,13^, or step-level public good games^14,15^), where a public good is produced as soon as at least a threshold of players in a group cooperates. For instance, in cooperative hunting^16,17^, a minimal number of encircling predators may be needed to successfully catch a prey. Similarly, in the collective defense of a common territory^18–20^, a minimal number of cooperating individuals may be needed to successfully defend the territory.

The explanation for social diversity and for the phenotypic plasticity of cooperative behavior then seems straightforward: cooperative behavior is less likely to evolve when the threshold is higher. Simply, one would expect that cooperation is less likely to evolve the more challenging is the production of the public good. E.g., when predators face a larger prey and a higher threshold of cooperating predators is therefore needed to successfully catch the prey, the evolution of cooperative hunting would be less likely; when the threat of intrusion is larger and more participation is thus needed to successfully defend a common territory, the defense of the common territory would be less likely to evolve. This paper shows that this intuition may not always be correct. It may be *because* producing a public good is challenging and requires a large number of cooperating players that cooperation can evolve. The reason is the following. If in any group of *n* players a threshold *k* of cooperators is needed to produce the public good, then from the perspective of a focal player the probability of this focal player’s participation ensuring that the threshold is exactly met (or: pivot probability), is maximal when a fraction $(k-1)/(n-1)$ of the population cooperates (such that the probability of the focal player being matched to exactly $(k-1)$ cooperators among $(n-1)$ other players is maximized). Yet, this maximal pivot probability itself is highest when the threshold is either low, or is high. In the same way, when drawing with replacement $(n-1)$ balls from an urn containing white and black balls, both the probability of drawing few white balls when the urn contains few white balls, and the probability of drawing many white balls when the urn contains many white balls, are large compared to the probability of drawing exactly $(n-1)/2$ white balls if the urn contains 50% white balls. For this reason, it may only be for the lowest and the highest thresholds that the gain in fitness of cooperating rather than defecting exceeds the participation costs, and that cooperation can evolve. Taking now participation costs sufficiently large such that cooperation cannot evolve with an intermediate threshold, lowering the threshold thus enables the evolution of cooperation, fitting the initial intuition. Yet, taking the same starting point, increasing the threshold and thus apparently making the evolution of cooperation more challenging, counter-intuitively encourages the evolution of cooperation.

From a broader perspective, this paper fits into a theoretical literature that explains the evolution of cooperation by considering non-linear as well as linear impact functions^21–28^ (where the impact function relates the level of the public good produced to the number of cooperating players in a group); for an overview, see^29^. In this way, cooperative games can not only take the form of an *n*-person version of the Prisoner’s Dilemma^7,30^ (where joint defection is the only stable fixed point), but can also be characterized by polymorphism (a stable fixed point exists where cooperators and defectors coexist), bistability (both a cooperative stable fixed point and a joint-defection stable fixed point exists), or a combination of both. Moreover, in this literature such game types are not studied in isolation, but it is explained how the shape of the impact function affects what game type is played. Yet, missing in this literature are predictions on how often we may expect cooperative situations to take on the form of individual game types. How often do we expect a combination of polymorphism and bistability, compared to situations with only polymorphism, only bistability, or situations that fit the Prisoner’s Dilemma? Our analysis answers this question by assessing the relative frequency of several game types across the parameter space, where the parameters we vary are the threshold and the participation costs. Because of the described effect where at any cooperative stable fixed point the probability of being pivotal is largest for the lowest and for the highest thresholds, across the parameter space, games where either few or many of the players cooperate are predicted to occur more frequently than games where an intermediate fraction of the players cooperates.

**Figure 1:**
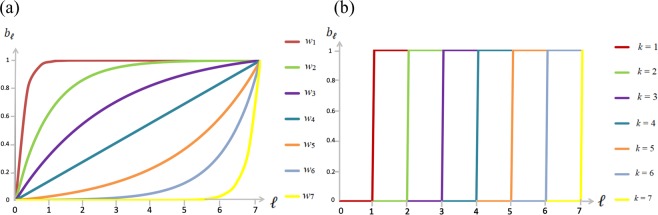
In part (**a**,**b**), each curve represents the impact function, i.e. production of the public good ${b}_{\ell }$ as a function of the number of cooperating players $\ell$ ranging from 0 cooperators to $n$ cooperators, where $n$ is the group size. Part (**a**) represents impact functions for several levels of the degree of complementarity *w* (with ${w}_{1} < {w}_{2} < {w}_{3} < {w}_{4} < {w}_{5} < {w}_{6} < {w}_{7}$), and (**b**) for several levels of threshold levels *k* (ranging from 1 to 7).

Within the literature on non-linear impact functions, most closely related to our analysis are models that vary the shape of the impact function by varying the degree of complementarity between the players’ cooperative efforts^26–28^. Figure 1 compares impact functions for several threshold levels (Fig. 1(b)), with impact functions for several degrees of complementarity (Fig. 1(a)). As is clear by comparing Fig. 1(b) to Fig. 1(a), the impact functions have the same shape with a minimal degree of complementarity as with the minimal threshold, and with a maximal degree of complementarity as with the maximal threshold. The difference is that for intermediate degrees of complementarity the impact function is closer to being linear, whereas for intermediate thresholds the impact function continues to take the form of a step function. This characteristic of the threshold impact function has two main advantages. First, it is realistic that impact functions are S-shaped, or so-called sigmoid functions^7^, where starting from a group with only defectors, the first cooperators in the group lead to increasingly large added benefits (accelerating part of the impact function), but the last cooperators add fewer and fewer benefits (decelerating part of the impact function). This contrasts with the impact functions depending on the degree of complementarity, which are either accelerating or decelerating, but never both. Threshold impact functions provide a good approximation for sigmoid functions, which are analytically hard to handle^7^. Moreover, the impact function may only approach continuous sigmoid functions for large groups, whereas for small groups a stepwise impact function is more realistic. A second advantage of threshold impact functions is that they account for a wider range of game types, in allowing for games that are characterized by both polymorphism and bistability^7,9^. This contrasts with impact functions with the degree of complementarity as a parameter, which for higher degrees of complementarity (accelerating impact functions) allow only for bistability but not for polymorphism (cooperation need not evolve, but if it does the entire population cooperates), and for lower degrees of complementarity (decelerating impact functions) allow only for polymorphism but not for bistability (cooperation always evolves, but the stable fixed point includes defectors as well as cooperators)^27,28^. In the threshold model, polymorphism without bistability is only allowed for the minimal threshold^31,32^, and bistability without polymorphism is only allowed for the maximal threshold^5^.

As pointed out by one of the referees, the effect of threshold increases we analyze is visible in numerical examples by Archetti and Scheuring^7^ (specifically their Fig. 2), both for threshold and for sigmoid impact functions, though the authors do not describe this effect in the text. The main message of this reference is to stress the existence of intermediate cooperative games where cooperators and defectors coexist, on top of more familiar extreme games where the population evolves to contain only defectors, or evolves to contain few cooperators. The contribution of our paper is twofold. First, for threshold public good games, we systematically analyze the effect of threshold increases on the level of cooperation that evolves. Second, we show that the level of cooperation may be low in the mentioned intermediate cooperative games, and that cooperation may even not evolve at all for intermediate thresholds, giving more weight across the parameter space to the extreme games.

**Figure 2:**
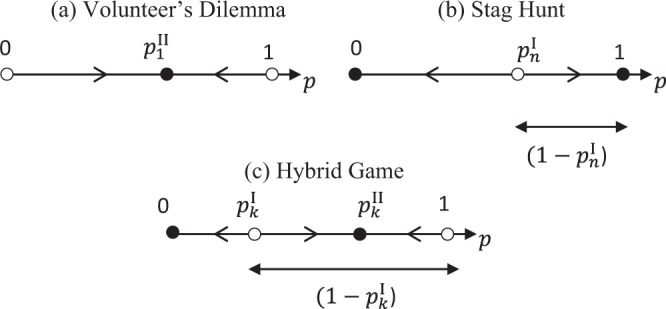
Phase portraits for cases where the threshold public-good game is a Volunteer’s Dilemma ($k=1$), a Stag Hunt $(k=n)$, and a Hybrid Game ($1 < k < n$) (where meant are *n*-player versions of these game types). A filled dot indicates a stable fixed point, a non-filled dot an unstable fixed point; the arrows on the axes indicate the direction in which the population evolves. ${p}_{k}^{{\rm{II}}}$ denotes the fraction of cooperators in a stable interior fixed point, ${p}_{k}^{{\rm{I}}}$ the fraction of cooperators in an unstable interior fixed point (with $1-{p}_{k}^{{\rm{I}}}$ equal to the size of the basin of attraction of a stable fixed point where at least a fraction of the players cooperates).

## Methods

We consider a well-mixed, infinitely large population of players that reproduces asexually. Players face the binary choice of cooperating or defecting. At any given moment, a fraction *p* of the population cooperates, and a fraction $(1-p)$ defects. Groups of *n* players are randomly and repeatedly formed. Denote by ${f}_{C}(p)$ the average fitness of cooperating, and by ${f}_{D}(p)$ the average fitness of defecting. Assuming that the fraction of cooperating players is determined by the continuous replicator dynamics^33^, the change over time of the fraction of cooperating players $\dot{p}$ equals:

21 $$\dot{p}=p(1-p)[{f}_{C}(p)-{f}_{D}(p)]$$

where

22 $${f}_{C}(p)=\mathop{\sum }\limits_{\ell =0}^{n-1}(\genfrac{}{}{0ex}{}{n-1}{\ell }){p}^{\ell }{(1-p)}^{n-1-\ell }{b}_{\ell +1}-c$$

23 $${f}_{D}(p)=\mathop{\sum }\limits_{\ell =0}^{n-1}(\genfrac{}{}{0ex}{}{n-1}{\ell }){p}^{\ell }{(1-p)}^{n-1-\ell }{b}_{\ell }.$$

In (2.2) and (2.3), $c$ denotes the participation costs (=costs of cooperating rather than defecting), and ${b}_{x}$ denotes the benefit the focal player obtains when exactly $x$ players in her group (including herself) cooperate. The focal player’s fitness when cooperating or defecting is determined by the number $\ell$ that cooperates among the $(n-1)$ other players in her group. Given the fraction $p$ of cooperating players in the population, the number of other cooperators in a group follows the binomial distribution, with the binomial coefficient $(\genfrac{}{}{0ex}{}{n-1}{\ell })=\frac{(n-1)!}{\ell !(n-1-\ell )!}$.

In order to specify the model as a threshold model, we follow existing notation in the literature^9^. Denote $k$ as the threshold, where $k\in \{1,2,\ldots ,n\}$. For the threshold public good game, we assume that ${b}_{x}=1$ when $x\ge k$ (meaning that the maximal value of the public good that can be produced is normalized to 1), and ${b}_{x}=0$ when $x < k$. Furthermore, we assume that $0 < c < 1$, meaning that a single player whose cooperation allows the group to meet the threshold, is better off cooperating. (2.2) and (2.3) can now be rewritten as

24 $${f}_{C}(p)=\mathop{\sum }\limits_{\ell =k-1}^{n-1}(\genfrac{}{}{0ex}{}{n-1}{\ell }){p}^{\ell }{(1-p)}^{n-1-\ell }-c$$

25 $${f}_{D}(p)=\mathop{\sum }\limits_{\ell =k}^{n-1}(\genfrac{}{}{0ex}{}{n-1}{\ell }){p}^{\ell }{(1-p)}^{n-1-\ell },$$

where ${f}_{D}(p)=0$ when $k=n$. It follows that the gains from switching from cooperating to defecting equal:

26 $${f}_{C}(p)-{f}_{D}(p)=(\genfrac{}{}{0ex}{}{n-1}{k-1}){p}^{k-1}{(1-p)}^{n-k}-c$$

Given the threshold nature of the game, when the focal player ends up in a group with either fewer than $(k-1)$ cooperators or more than $(k-1)$ cooperators, the benefit part of the gains from switching equals zero. The benefit part of the gains from switching therefore equals the probability of being pivotal in one’s group (or in short: *pivot probability*), times the benefit of being pivotal; as the latter is normalized to 1, the benefit part of the gains from switching equals the pivot probability itself, which we denote as ${\pi }_{k}(p)=(\genfrac{}{}{0ex}{}{n-1}{k-1}){p}^{k-1}{(1-p)}^{n-k}$. By (2.1), *p* = 0 and *p* = 1 are always fixed points; by (2.6), interior points may additionally exist for *p* such that ${\pi }_{k}(p)=c$. The existence of such interior fixed points depends on the properties of ${\pi }_{k}(p)$.

## Results

The following properties of ${\pi }_{k}(p)$ depending on $k$ are easily checked, where Property 1(i) and 1(ii) have been previously derived in the literature^9^ (all proofs of the properties and results in this paper can be found in part A of the Supporting Information):

**Property 1 about** $${{\boldsymbol{\pi }}}_{{\boldsymbol{k}}}({\boldsymbol{p}})$$ **:**

(i) For $k=1$, ${\pi }_{k}(p)$ is a decreasing function with ${\pi }_{k}(0)=1$ and ${\pi }_{k}(1)=0$.
(ii) For $k\in \{2,3,\ldots ,n-1\}$, ${\pi }_{k}(p)$ is a unimodal function that reaches a maximum for $p=\frac{k-1}{n-1}$, increases for $p < \frac{k-1}{n-1}$, but decreases for $p > \frac{k-1}{n-1}$, and for which ${\pi }_{k}(0)={\pi }_{k}(1)=0$ and $0 < {\pi }_{k}\left(\frac{k-1}{n-1}\right) < 1$.
(iii) For $k=n$, ${\pi }_{k}(p)$ is an increasing function with ${\pi }_{k}(0)=0$ and ${\pi }_{k}(1)=1$.

Property 1 can be understood by the fact that with a given threshold *k*, the focal player is most likely to be pivotal if the fraction of cooperating players within the population is $\frac{k-1}{n-1}$. Result 1 now follows directly from Property 1, and derives the stable fixed points as a function of the participation costs and of the threshold level (where the parts of Result 1 for $1\le k < n$ have been stated elsewhere^7,9,29^). The result can be concisely stated by looking at the type of the game played as a function of the parameters; these game types, and their corresponding phase diagrams are represented in Fig. 2. These game types have originally been defined for, and are most often described in the context of two-player games; when we refer to these games here, we mean *n*-player versions of them^4,5,7,32,34,35^, where we omit the qualifier “*n*-player” for brevity. A Snowdrift Game^36^ is a game with a single interior stable fixed point where a fraction of the players cooperates (in particular, as in all cases where the game is a Snowdrift Game, a single cooperator suffices to produce the public good, the game is a Volunteer’s Dilemma^37^). A Prisoner’s Dilemma is a game with a single stable fixed point where all players defect^30^. A Stag Hunt^34^ is a game with both a stable fixed point where the entire population cooperates, and a stable fixed point where the entire population defects. A Hybrid Game shares features of the Volunteer’s Dilemma and the Stag Hunt, and has both a stable fixed point where all players defect (which we refer to as the defective stable fixed point), and an interior cooperative stable fixed point.

We first look at non-interior stable fixed points. While in existing literature the focus is on the cooperative stable fixed point^9^, we include in our analysis the possibility that *p* = 0 is a stable fixed point. In fact, such a defective stable fixed point exists for all $k > 1$, as it is then a best response to defect if the entire population defects. For $k=1$, however, if the entire population defects, the individual player is better off cooperating, and $p=0$ is an unstable fixed point. Additionally, for $k=n$, $p=1$ is a stable fixed point, because the individual player is then better off cooperating in a population where all players cooperate; however, for $k < n$, the individual player is better off defecting in such a case, and $p=1$ is an unstable fixed point.

We next look at interior stable fixed points. For $1 < k < n$, Property 1(ii) tells us that ${\pi }_{k}(p)$ is hill-shaped, such that for participation costs that are not too large, two interior fixed points exist. For $k=1$ (respectively $k=n$), given that ${\pi }_{k}(p)$ is decreasing (increasing) and ranges from 1 to 0 (from 0 to 1), a single interior fixed point always exists. Denote in particular by ${p}_{k}^{{\rm{I}}}$ an interior fixed point with ${\pi }_{k}({p}_{k}^{{\rm{I}}})=c$ in the increasing part of ${\pi }_{k}(p)$, and by ${p}_{k}^{{\rm{II}}}$ an interior fixed point with ${\pi }_{k}({p}_{k}^{{\rm{II}}})=c$ in the decreasing part of ${\pi }_{k}(p)$. Clearly, any fixed point ${p}_{k}^{{\rm{I}}}$ is unstable, because for a slightly larger (smaller) fraction of cooperating players, the individual player is better off cooperating (defecting). Summarizing, for $k=1$, the game has a single interior cooperative fixed point. Also, for $1 < k\le n$, the game has two stable fixed points, namely the defective stable fixed point, and the cooperative stable fixed point. In this case, we consider the size of the basin of attraction of the cooperative stable fixed point, i.e. $(1-{p}_{k}^{{\rm{I}}})$, as the probability that this fixed point will evolve. The underlying reasoning is that each initial population is equally likely, so that the probability that the cooperative stable fixed point evolves is equal to the probability that the initial population lies in its basin of attraction.

In order to describe Result 1, we define ${\bar{c}}_{k}={\pi }_{k}\left(\frac{k-1}{n-1}\right)=(\genfrac{}{}{0ex}{}{n-1}{k-1})\frac{{(k-1)}^{k-1}{(n-k)}^{n-k}}{{(n-1)}^{n-1}}$ as the maximal pivot probability as a function of $p$, where by Property 1 ${\bar{c}}_{0}={\bar{c}}_{n}=1$, and where $0 < {\bar{c}}_{k} < 1$ for $1 < k < n$.

**Result 1. Types of games played as a function of thresholds and participation costs**.

(i) For the minimal threshold ($k=1$), the game has a unique interior fixed point where a fraction ${p}_{1}^{{\rm{II}}}=1-{c}^{1/(n-1)}$ of players cooperates (**Volunteer’s Dilemma**).
(ii) For intermediate thresholds ($1 < k < n$), when participation costs are large ($c > {\bar{c}}_{k}$), the game has a unique stable fixed point where all players defect ($p=0$) (**Prisoner’s Dilemma**). When participation costs are small ($c < {\bar{c}}_{k}$), the game both has a fixed point where all players defect ($p=0$ and a stable fixed point where a fraction ${p}_{k}^{{\rm{II}}}$ of the players cooperates, with $0 < {p}_{k}^{{\rm{II}}} < 1$ (where ${p}_{k}^{{\rm{II}}}$ is implicitly given by ${\pi }_{k}({p}_{k}^{{\rm{II}}})=c$) (**Hybrid Game**). The former stable fixed point has a basin of attraction ${p}_{k}^{{\rm{I}}}$, the latter stable fixed point a basin of attraction $(1-{p}_{k}^{{\rm{I}}})$ (where ${p}_{k}^{{\rm{I}}}$ is implicitly given by ${\pi }_{k}({p}_{k}^{{\rm{I}}})=c$, and where ${p}_{k}^{{\rm{I}}} < {p}_{k}^{{\rm{II}}}$).
(iii) For the maximal threshold ($k=n$), the game both has a stable fixed point where all players defect ($p=0$), and a stable fixed point where all players cooperate ($p=1$) (**Stag Hunt**). The former stable fixed point has a basin of attraction ${p}_{n}^{{\rm{I}}}$, the latter stable fixed point a basin of attraction $(1-{p}_{n}^{{\rm{I}}})$, where ${p}_{n}^{{\rm{I}}}={c}^{1/(n-1)}$.

**Figure 3:**
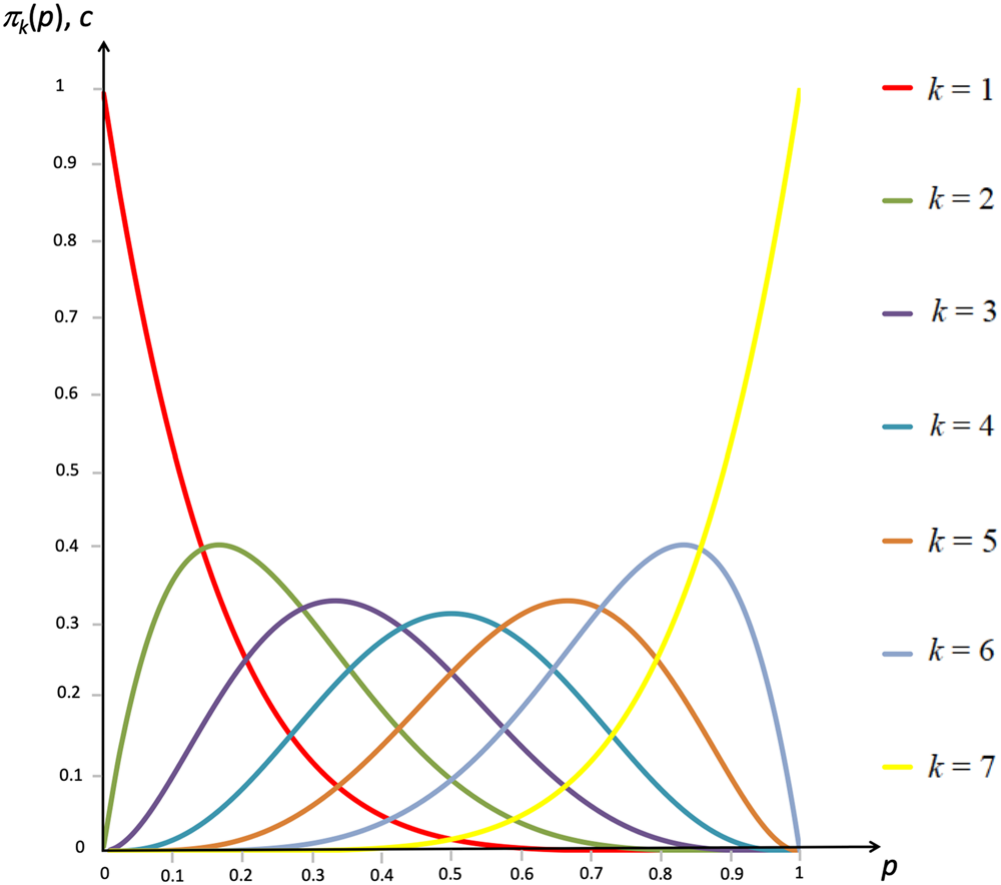
Pivot probabilities ${\pi }_{k}(p)$ for all nonzero threshold levels *k* in the case of groups with size $n=7$. By fixing a level for the participation costs *c* along the Y-axis and looking for the fixed points, one can see how the type of the game changes as a function of the threshold.

In order to look at the effect of increases in the threshold, we now derive Property 2 about ${\pi }_{k}(p)$, which looks at how the shape of the pivot probability ${\pi }_{k}(p)$ is affected by the threshold. An example is given in Fig. 3 for $n=7$. Property 2 can be understood as follows. An increase in the threshold means that the pivot probability becomes smaller for a lower range of fractions of cooperation players, and larger for an upper range of such fractions, and that the fraction of cooperating players for which the pivot probability is maximal increases (Property 2(i)). At the same time, the maximal pivot probability itself is a U-shaped function of the threshold (Property 2(ii)); this is because the threshold is most likely to be exactly achieved when the threshold is low and a small fraction of the players cooperates, or when the threshold is high and a large fraction of the players cooperates. Furthermore, Property 2(iii) establishes some symmetry properties of the pivot probabilities, and Property 2(iv) calculates the maximal pivot probabilities whenever possible.

**Property 2 about** $${{\boldsymbol{\pi }}}_{{\boldsymbol{k}}}({\boldsymbol{p}})$$ **:**

(i) ${\pi }_{k}(p)\gtreqqless{\pi }_{k+1}(p)$ iff $p\lesseqqgtr\frac{k}{n}$. Combine this fact with Property 1(ii), and consider $p\in \left[\frac{k-1}{n-1},\frac{k}{n-1}\right]$. Then for $p=\frac{k-1}{n-1}$, ${\pi }_{k}(p)$ reaches a maximum, ${\pi }_{k}(p) > {\pi }_{k+1}(p)$, and ${\pi }_{k+1}(p)$ increases; for $\frac{k-1}{n-1} < p < \frac{k}{n}$, ${\pi }_{k}(p) > {\pi }_{k+1}(p)$, ${\pi }_{k}(p)$ decreases and ${\pi }_{k+1}(p)$ increases; for $p=\frac{k}{n}$, ${\pi }_{k}(p)={\pi }_{k+1}(p)$; for $\frac{k}{n} < p < \frac{k}{n-1}$, ${\pi }_{k}(p) < {\pi }_{k+1}(p)$, ${\pi }_{k}(p)$ decreases and ${\pi }_{k+1}(p)$ increases; for $p=\frac{k}{n-1}$, ${\pi }_{k}(p) < {\pi }_{k+1}(p)$, ${\pi }_{k}(p)$ decreases and ${\pi }_{k+1}(p)$ reaches a maximum.
(ii) ${\bar{c}}_{k} > {\bar{c}}_{k+1}$ for $k < \frac{n+1}{2}$, and ${\bar{c}}_{k} < {\bar{c}}_{k+1}$ for $k > \frac{n+1}{2}$.
(iii) $${p}_{x}^{{\rm{II}}}(c)=1-{p}_{n-x+1}^{{\rm{I}}}(c)$$, and $${\bar{c}}_{x}={\bar{c}}_{n-x+1}$$
(iv) ${\bar{c}}_{1}={\bar{c}}_{n}=1$. Furthermore, ${\bar{c}}_{2}={\bar{c}}_{n-1}={\left(\frac{n-2}{n-1}\right)}^{n-2}$, which equals 0.5 for $n=3$, and converges to *e*^−1^ for large *n*. For odd *n*, $\,{\bar{c}}_{(n+1)/2}$ equals 0.5 for *n* = 3 and converges to 0 for large *n*; for even *n*, ${\bar{c}}_{(n+2)/2}={\bar{c}}_{n/2}$ equals 4/9 for $n=4$ and converges to 0 for large *n*.

We now come to our main result in Result 2, which follows directly from Property 2 and looks at how, for given levels of the participation costs, the game type changes as a function of the threshold. As the level of cooperation that can evolve differs across game types (see Fig. 2), these game-changing effects of increases in the threshold can be interpreted as effects on the level of cooperation. In detail, Result 2 shows that for sufficiently large participation costs (Result 2(i) and 2(ii)), increasing the threshold has a U-shaped effect on the level of cooperation, in that cooperation can evolve both for low and for high thresholds, but not for intermediate thresholds. Put otherwise, for sufficiently large participation costs, we have a Volunteer’s Dilemma or Hybrid Game for low thresholds and a Stag Hunt or Hybrid game for high thresholds (in which cases cooperation can evolve), but a Prisoner’s Dilemma for intermediate thresholds (in which cooperation cannot evolve).

**Result 2. game-changing effects of an increase in the threshold**.

(i) For the upper range of large participation costs (for ${\bar{c}}_{2}={\bar{c}}_{n-1} < c < 1$), the game is a Volunteer’s Dilemma with the minimal threshold ($k=1$), a Prisoner’s Dilemma with intermediate thresholds ($1 < k < n$), and a Stag Hunt with the maximal threshold ($k=n$).
(ii) For the lower range of large participation costs (for ${\bar{c}}_{(n+1)/2} < c < {\bar{c}}_{2}={\bar{c}}_{n-1}$ when $n$ is odd, and for ${\bar{c}}_{(n+1)/2}={\bar{c}}_{n/2} < c < {\bar{c}}_{2}={\bar{c}}_{n-1}$ when $n$ is even), the game is a Volunteer’s Dilemma with the minimal threshold ($k=1$). As the threshold is now increased, the game successively becomes a Hybrid Game, then a Prisoner’s Dilemma, and then again a Hybrid game. With the maximal threshold ($k=n$), the game is a Stag Hunt.
(iii) For small participation costs (for $0 < c < {\bar{c}}_{(n+1)/2}$ when $n$ is odd, and for $0 < c < {\bar{c}}_{(n+1)/2}={\bar{c}}_{n/2}$ when $n$ is even), the game is a Volunteer’s Dilemma with the minimal threshold ($k=1$), a Hybrid Game with intermediate thresholds ($1 < k < n$), and a Stag Hunt with the maximal threshold ($k=n$).

The interpretation of the game-changing effects in Result 2(i) and 2(ii) as effects on the level of cooperation is a strong interpretation, as it applies for all formal measures of the level of cooperation that one may consider. For instance, one may simply measure the level of cooperation by the fraction of cooperating players in the cooperative stable fixed point^9^. Yet, a more goal-oriented measure may be found by calculating the probability that the public good is produced within any individual group; moreover, one may include in this measure the expected participation costs, so that one considers the expected payoff that each individual player obtains. Finally, for each of these measures, one may additionally include the ex-ante probability that cooperation evolves at all, as given by the basin of attraction of the cooperative stable fixed point. Whichever of these formal measures one considers, as the game is a Prisoner’s Dilemma for intermediate thresholds and has no cooperation, the U-shape effect is maintained.

It is natural to ask oneself whether the U-shaped effect on the level of cooperation implied by Result 2(i) and 2(ii) is maintained when one does not only consider game-changing effects of higher thresholds, but also looks at the effect on the level of cooperation when a higher threshold does not change the type of the game. As shown in part B of the Supporting Information, for a range of participation costs just below the maximal pivot probability, the effect of the threshold on the level of cooperation remains U-shaped, no matter what measure for the level of cooperation one considers. Moreover, by the hysteresis effect^3,6^, the game-changing effects in Result 2(i) may extend to smaller participation costs, where for intermediate thresholds the defective fixed point would continue to be the only outcome that can evolve. The point of the hysteresis effect is that for participation costs just below the maximal pivot probability, the fraction of cooperating players in the cooperative stable fixed point is only slightly above the critical fraction of cooperating players that brings one in the basin of attraction of this stable fixed point. Thus, a small shock in the fraction of cooperating players can lead to joint defection evolving. Once joint defection has evolved, a small shock in the fraction of cooperating players cannot bring one back to the cooperative stable fixed point. In the same way, for participation costs just below the maximal pivot probability, a small shock to the participation costs can turn the game into a Prisoner’s Dilemma. Once joint defection has evolved, a return to the original participation costs does not allow the cooperative stable fixed point to evolve again. For this reason, Result 2(i) may effectively apply for a wider range of participation costs. Yet, for the smallest participation costs, as is clear from Fig. 3, the hysteresis effect does not apply, because participation costs are far below the maximal pivot probability, and because the fraction of cooperators in the cooperative stable fixed point is much larger than the critical fraction dividing the basins of attraction. As shown in part B of the Supporting Information, the effect of the threshold on the level of cooperation is ambiguous in this case, and depends on the precise measure of the level of cooperation that is considered.

**Figure 4:**
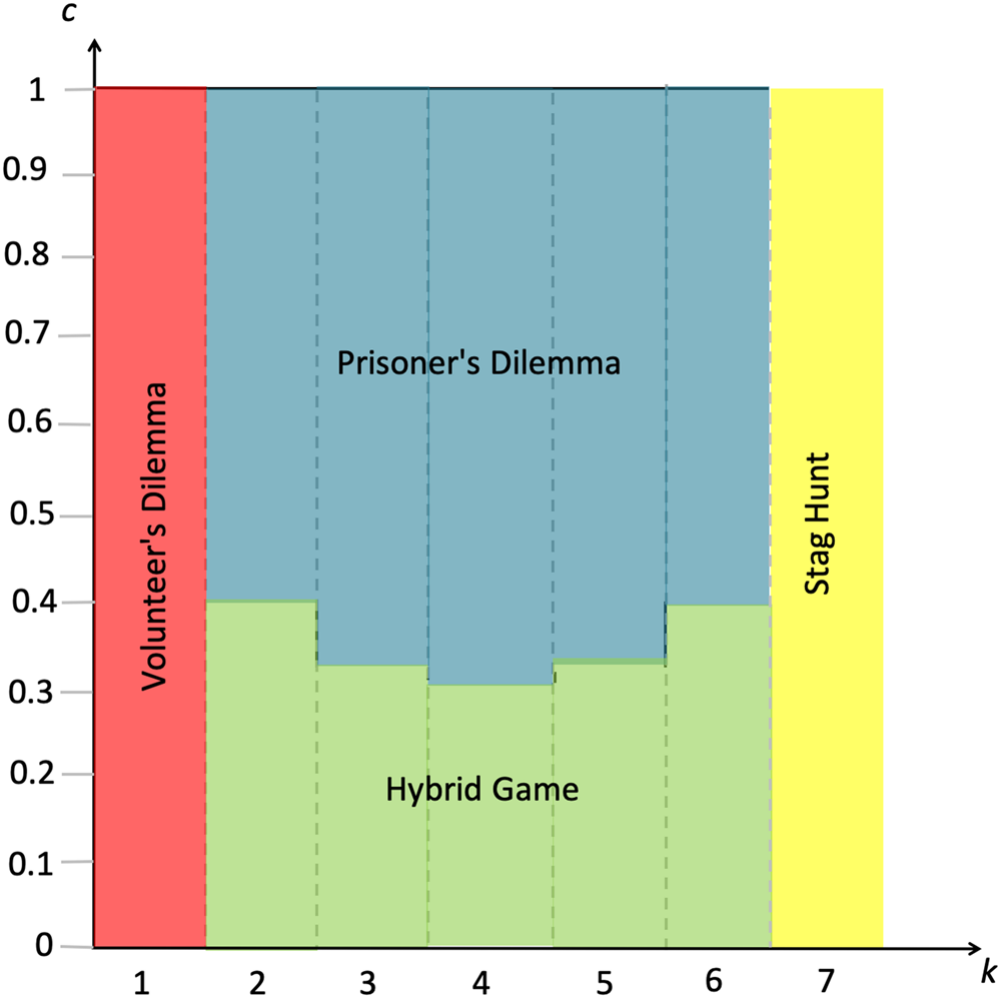
Parameter space representing all possible participation costs (vertical axis), and all possible thresholds (horizontal axis) for the case *n* = 7. As thresholds are natural numbers, they are represented as bars. The figure represents the incidence of the several game types (see Fig. 2) across the parameter space, where to each color corresponds a game type (where the game types refer to *n*-player versions of these games). As shown in the figure, the game types that allow for the evolution of cooperation (all other games than the Prisoner’s Dilemma) take up a large part of the parameter space for lower, and for higher thresholds. For large participation costs, the only game types that allow for the evolution of cooperation are the Volunteer’s Dilemma and the Stag Hunt.

Another way to interpret our results is in terms of the predicted frequency of the different game types. What game types receive more weight across the parameter space covering all possible combinations of participation costs and thresholds? To assess this, Fig. 4 represents for the example $n=7$ all possible thresholds ($k=1,2,\ldots ,7$) on the X-axis, and all participation costs ($0 < c < 1$) on the Y-axis; as a function of these, the type of the game played is indicated. As is clear from Fig. 4, roughly, games in which the evolution of cooperation is possible apply for smaller participation costs (as the Prisoner’s Dilemma applies for larger participation costs). Yet, both for low and for high thresholds, participation costs need to be less small to allow for the evolution of cooperation, with such evolution possible over the entire participation cost range both for the minimal threshold (in which case the game is a Volunteer’s Dilemma) and the maximal threshold (in which case the game is a Stag Hunt). For this reason, across the parameter space, among the games that allow for the evolution of cooperation, games with low and with high thresholds have more weight. This effect is particularly pronounced for small groups. For instance, assuming a uniform distribution of both *k* and *c*, applying Property 2(iv), it can be checked that with *n* = 3 the Snowdrift game and the Stag Hunt each take up 1/3 of the parameter space, whereas the Hybrid game and the Prisoner’s Dilemma each take up 1/6; with *n* = 4 the Snowdrift game and the Stag Hunt each take up 25% of the parameter space, the Hybrid game takes up 22%, and the Prisoner’s Dilemma takes up 28%. For larger groups, Hybrid games take up a larger part of the parameter space; yet within the class of Hybrid games itself, those where few or where many players cooperate in the cooperative stable fixed point again receive more weight, as is clear from Fig. 4.

We further look at how the picture in Fig. 4 is affected by group size. Given Property 2(iv), as ${\bar{c}}_{(n+1)/2}$ approaches 0 for large *n* when *n* is odd (and as ${\bar{c}}_{(n+2)/2}={\bar{c}}_{n/2}$ approach 0 for large $n$ when $n$ is even), the U-shaped effect of the threshold on the level of cooperation in Results 2(i) and 2(ii) applies for a wider range of participation costs the larger the group size. Yet, at the same time, it is clear that for large *n* the share of the parameter space taken up by the Volunteer’s Dilemma and the Stag Hunt becomes smaller and smaller. Moreover, the fraction of cooperating players in the unique stable fixed point of the Volunteer’s Dilemma, and the basin of attraction of the joint-cooperation fixed point in the Stag Hunt, get smaller and smaller as *n* is increased. This means that for the U-shaped effect in Result 2(i), the level of cooperation for the minimal and maximal threshold is small, so that the U-shaped effect is weak. While for group sizes approaching infinity the fixed points can be calculated, these appear less relevant because the Prisoner’s Dilemma becomes dominant, and in the remaining cases where cooperation can evolve, the predicted level of cooperation is vanishingly small. Overall, our two results about the counter-intuitive effect of a higher threshold and about the frequency of different game types across the parameter space, are thus most pronounced for smaller groups.

## Discussion

The first conclusion of this paper, namely that a higher threshold for the successful production of a public good may enable rather than hamper the evolution of cooperation, can be linked to a hypothesis that dates back to at least Kropotkin^38,39^, stating that the evolution of cooperation takes place when harsh environments make it in organisms’ interest to cooperate. This hypothesis may be termed the common-enemy hypothesis, with a harsher environment acting as a common enemy encouraging cooperation. The hypothesis forms an alternative to more well-known explanations for the evolution of cooperation, including direct^40^ and indirect reciprocity^41^, network reciprocity^42^, kin selection^43^, and group selection^44^. In our analysis, a harsh environment takes the form of e.g. a larger prey, such that more cooperating predators are needed to successfully catch a prey, or of a higher threat of intrusion, such that a larger number of defenders is needed to successfully protect a common territory.

Existing theoretical underpinnings for the common-enemy hypothesis argue that in harsher environments, players who defect from cooperation become to a larger extent the victim of their own defection (the so-called boomerang effect)^27,28,45^. Put otherwise, a harsher environment makes each player’s cooperative effort to a higher extent pivotal, and thus changes the benefit of being pivotal. The argument is therefore that harsher environments change the shape of the impact function of the public good produced by the players, and in particular makes the impact function to a larger extent accelerating, such that the last player who cooperates contributes more (see Fig. 1(a)). In the current paper, a harsher environment does not change the shape of the impact function, in the sense that the player whose cooperative effort ensures that the threshold is reached, always contributes the full value of the public good (the impact function always takes the shape of a step function; see Fig. 1(b)). Instead, a harsher environment in the form of a larger threshold means that the probability of the individual player being pivotal in her group becomes larger, so that a cooperative stable fixed point may come to exist, where none existed before. In the current paper, it is therefore not the *benefit* of being pivotal that is affected by the harshness of the environment, but the *probability* of being pivotal.

The second conclusion from this paper is that cooperation where either a small fraction in any group cooperates, or a large fraction, should evolve more frequently than cooperation where an intermediate fraction of a group cooperates. When modeling the evolution of cooperation, evolutionary game theory has often focused on stylized games such as the Volunteer’s Dilemma (leading to polymorphism) or the Stag Hunt (leading to bistability)^7^. While this focus is due to the simplifying assumption of two-player games, which do not allow for Hybrid games characterized by both polymorphism and bistability, our analysis suggests that this focus has some merit, given our prediction that cooperation where either a small, or a large fraction of a group cooperates, evolves more frequently. It is tempting to see a similar pattern in canonical examples of cooperative behavior, which often include sentinel behaviour, where a single sentinel may suffice to produce the public good^46^, as well as cooperative hunting, where several hunters need to cooperate to catch the prey^16,17^. However, a systematic study looking at the relevance of several game types across a broad range of cooperative instances is missing, and furthermore, cooperative hunting has itself been observed to be characterized by polymorphism, with both cooperators and defectors^16^.

Our results can be linked to the literature that studies the effect of group size on the probability of cooperation, connected to the so-called Olson conjecture^9,47^, stating that cooperation is hampered by larger group sizes. Given that a cooperative stable fixed point exists over a large range of participation costs when the threshold approaches the group size, it follows that holding the threshold fixed and decreasing group size until it approaches the threshold can enable the evolution of cooperation – which is fully in line with the Olson conjecture. If group size is itself subject to evolution^48^, it is therefore possible that groups evolve to be equal in size to the threshold needed to achieve successful public-good production. Yet, in a similar way, one could reason that, since for low thresholds it is also the case that a cooperative stable fixed points exists for a large range of participation costs, starting from a fixed intermediate threshold, increasing group size could also enable the evolution of cooperation, as the fixed threshold then becomes small compared to group size. However, this reasoning is wrong, as is clear by looking at the effect of group size on pivot probabilities^9^. The point is that, while by decreasing group size one can always ensure that the threshold becomes equal to the group size (so that the game becomes a Stag Hunt), by increasing group size for a given threshold, even though the threshold may become small compared to group size, one can never make the threshold equal to 1 (so that the game never becomes a Volunteer’s Dilemma). Our results are thus in line with the Olson conjecture, in that all else equal an increase in group size is still predicted to hamper the evolution of cooperation. Still, our results imply that, if for a given group size and threshold smaller than the group size cooperation cannot evolve, then increasing the group size *and* at the same time increasing the threshold even more so that it approaches the new group size, can still enable the evolution of cooperation. This is because of the large pivot probabilities that can be achieved if the threshold approaches the group size.

An avenue for future research is to study the common-enemy hypothesis in combination with standard explanations for the evolution of cooperation, such as (in)direct reciprocity or network reciprocity. Notably, in statistical physics (for an overview, see^49^) the effect of threshold changes on the evolution of cooperation has been studied for structured populations^50^. Because of network reciprocity, in simulations, an opposite effect to the one we describe for sufficiently large participation costs is observed, where the level of cooperation that evolves is higher for intermediate thresholds than for low or high thresholds. The intuition for this result is that it is for intermediate thresholds that cooperators benefit most from the vicinity of other cooperators. For a similar reason, cooperation can also be higher for intermediate group sizes in case of network reciprocity^51^. As a well-mixed population can be considered as a limit case of a structured population^52^, a question for future research is therefore for what population structures the effect we describe applies, and for what population structures an opposite effect applies.

## Supplementary information

Supplementary information.
