# Supplementary material for: High thresholds encouraging the evolution of cooperation in threshold public-good games
Source: Sci Rep. 2020 Apr 3;10:5863. doi: 10.1038/s41598-020-62626-3 (PMC7125178; doi:10.1038/s41598-020-62626-3)
Supplement: Supplementary file 1 — Supplementary information. [file 41598_2020_62626_MOESM1_ESM.pdf]

Supplementary Information to "High thresholds encouraging the evolution of cooperation in threshold public-good games", Kris De Jaegher.

Part A: proofs.

Proof of Property 1:

$$\frac{\partial}{\partial p}\binom{n-1}{k-1}p^{k-1}(1-p)^{n-k} =$$
$$\binom{n-1}{k-1}[(k-1)p^{k-2}(1-p)^{n-k}-(n-k)p^{k-1}(1-p)^{n-k-1}] =$$
$$\binom{n-1}{k-1}p^{k-2}(1-p)^{n-k-1}[(k-1)-(n-1)p]$$

It follows that with $1<k<n$, $\pi_k(p)$ increases for $p<\frac{k-1}{n-1}$, decreases for $p>\frac{k-1}{n-1}$, and reaches a maximum for $p=\frac{k-1}{n-1}$. It is the case that $0<\bar{c}_k=\pi_k\left(\frac{k-1}{n-1}\right)<1$, as the pivot probability can never reach 1 in a heterogeneous population. With $k=1$, the derivative is negative, and with $k=n$ it is positive.
QED

Proof of Result 1:
(i) Given Property 1(i), as $\pi_k(p)$ is a decreasing function ranging from 1 to 0, and as $0<c<1$, there is a single p_1^{II} such that $\pi_k(p_1^{\text{II}})=\binom{n-1}{0}(1-p_1^{\text{II}})^{n-1}=c$, so that $p_1^{\text{II}}=1-c^{1/(n-1)}$. As $\pi_k(p)$ is decreasing, this is the unique stable fixed point.
(ii) Given Property 1(ii), as $\pi_k(p)$ increases in p for low p, increases in p for high p, and as $\bar{c}_k<1$, $\pi_k(p)=c$ has two solutions p_k^{I} and p_k^{II} for $c<\bar{c}_k$. Given that $\pi_k(p)>c$ for $p_k^{\text{I}}<p<p_k^{\text{II}}$, and that $\pi_k(p)<c$ for $p<p_k^{\text{I}}$ and $p>p_k^{\text{II}}$, it follows that p_1^{II} is an stable fixed point, and p_k^{I} is an unstable fixed point. It follows that the two stable fixed points are $p=0$ and $p=p_k^{\text{II}}$. Given that the fraction of cooperating players increases for $p>p_k^{\text{I}}$, it follows that $1-p_k^{\text{I}}$ is the basin of attraction of stable fixed point $p=p_1^{\text{II}}$.
(iii) Given Property 1(iii), as $\pi_k(p)$ is an increasing function ranging from 0 to 1, and as $0<c<1$, there is a single p_1^{I} such that $\pi_k(p_1^{\text{I}})=\binom{n-1}{n-1}(p_1^{\text{I}})^{n-1}=c$, so that $p_1^{\text{I}}=c^{1/(n-1)}$. As $\pi_k(p)$ is increasing, this is an unstable fixed point, and the stable fixed points are $p=0$ and $p=1$.
QED

Proof of Property 2:
(i)
$$\pi_k(p)=\binom{n-1}{k-1}p^{k-1}(1-p)^{n-k} \gtreqless \pi_{k+1}(p)=\binom{n-1}{k}p^{k}(1-p)^{n-k-1}$$
iff
$$\frac{(n-1)!}{(k-1)!(n-k)!}p^{k-1}(1-p)^{n-k} \gtreqless \frac{(n-1)!}{k!(n-1-k)!}p^{k}(1-p)^{n-k-1}$$
iff
$$k(1-p) \gtreqless (n-k)p$$

iff $p \lesseqgtr \frac{k}{n}$.
By Property 1(i), $\pi_k(p)$ reaches a maximum at $\frac{k-1}{n-1}$, and $\pi_{k+1}(p)$ at $\frac{k}{n-1}$. The property now follows by the fact that $\frac{k-1}{n-1} < \frac{k}{n} < \frac{k}{n-1}$.
(ii)
$\bar{c}_k = \binom{n-1}{k-1} \frac{(k-1)^{k-1}(n-k)^{n-k}}{(n-1)^{n-1}} \gtreqless \bar{c}_{k+1} = \binom{n-1}{k} \frac{k^k(n-1-k)^{n-1-k}}{(n-1)^{n-1}}$
iff
$\frac{(n-1)!}{(k-1)!(n-k)!}(k-1)^{k-1}(n-k)^{n-k} \gtreqless \frac{(n-1)!}{k!(n-1-k)!}k^k(n-1-k)^{n-1-k}$
iff
$\frac{1}{(k-1)!(n-1-k)!}(k-1)^{k-1}(n-k)^{n-1-k} \gtreqless \frac{1}{(k-1)!(n-1-k)!}k^{k-1}(n-1-k)^{n-1-k}$
iff
$\left(\frac{k-1}{k}\right)^{k-1} \gtreqless \left(\frac{n-1-k}{n-k}\right)^{n-1-k}$

Note that these two expressions are equal for $k = n/2$. Note that if $k = k^*$ in the left-hand side, then the right-hand side takes on the same value if $k = n - k^*$. As the left-hand side is a decreasing function of k, it follows that the right-hand side is an increasing function of k, whatever n. Therefore, $\bar{c}_k \gtreqless \bar{c}_{k+1}$ iff $k \lesseqgtr (n+1)/2$.
(iii)
For any fixed point $p = p^*$ for a given threshold $k = k^*$, it is the case that $\binom{n-1}{k^*-1} p^{*k^*-1}(1-p^*)^{n-k^*,} = c$. We show that $p = 1 - p^*$ is then a fixed point for threshold $k = n - k^* + 1$. This is the case iff $\binom{n-1}{n-k^*}(1-p^*)^{n-k^*}(p^*)^{k^*-1} = c$. This is indeed the case, as $\binom{n-1}{k^*-1} = \binom{n-1}{n-k^*}$. Similarly, consider $k = k^*$, in which case $\bar{c}_{k^*} = \binom{n-1}{k^*-1} \frac{(k^*-1)^{k^*-1}(n-k^*)^{n-k^*}}{(n-1)^{n-1}}$. Consider $k = n - k^* + 1$, in which case $\bar{c}_{n-k^*+1} = \binom{n-1}{n-k^*} \frac{(n-k^*)^{n-k^*}(k^*-1)^{k^*-1}}{(n-1)^{n-1}}$.
(iv)
This can be directly calculated.
QED

Proof of Result 2:
This follows directly from combining Result 1 and Property 2.

Part B: extension to small participation costs and non-game changing effects

We consecutively treat several measures of the level of cooperation, where each time we start with results for the effect of a higher threshold on the measure that can be generally shown, and next look at the specific example with groups of 7 players, in line with Figure 3 in the body of the paper. This example looks at the effect of the threshold on the measures for several participation cost levels, which include cost levels just below the maximal pivot probabilities. The measures under B1, B4 and B5 look at the level of cooperation within a cooperative stable fixed point. The rest of the measures take into account the basin of attraction of the cooperative stable fixed point, and include the probability that a cooperative stable fixed point evolves in the first place. All measures are a function of $p_k^{\mathrm{I}}(c)$ and $p_k^{\mathrm{II}}(c)$, which represent the two possible interior fixed points as a function of participation costs c. While for all measures, the U-shaped effect of higher thresholds in Result 2 in the body of the paper applies for sufficiently large participation costs, for smaller participation costs the form of the effect depends on the measure considered.

B1. Fraction of cooperating players in cooperative stable fixed point

The measure considered here is $p_k^{\mathrm{II}}(c)$, namely the fraction of cooperating players if the players achieve a cooperative stable fixed point.

Result B1: if a cooperative stable fixed point exists for both k_1 and k_1+1, then $p_{k_1+1}^{\mathrm{II}}(c) > p_{k_1}^{\mathrm{II}}(c)$.
Proof:
By Property 2(i), for two thresholds k_1 and k_1+1, $\pi_{k_1}(p)$ and $\pi_{k_1+1}(p)$ intersect for a p_1 such that $\pi_{k_1}(p_1)$ decreases and $\pi_{k_1+1}(p_1)$ increases, where $\pi_{k_1}(p) > \pi_{k_1+1}(p)$ for $p < p_1$ and $\pi_{k_1}(p) < \pi_{k_1+1}(p)$ for $p > p_1$. The result follows directly from this fact.
QED

For the case $k = 7$, Figure A1 represents p_k^{II} as a function of k for several levels of the participation costs, where for ease of representation the levels of p_k^{II} are connected by lines. The non-monotonic effects for larger participation costs are only obtained because the game becomes a Prisoner's Dilemma; as long as after an increase in the threshold there continues to be an interior fixed point, the effect is monotonically increasing.

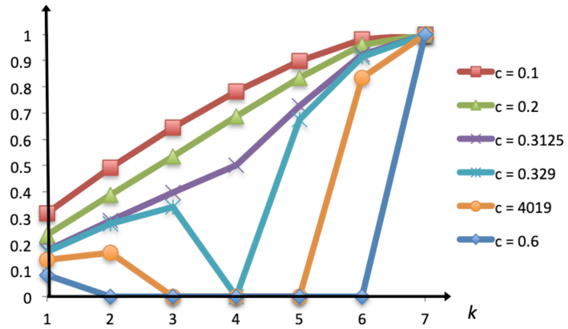

Figure B1. Effect of the threshold on the fraction of cooperating players in the cooperative stable fixed point for several participation cost levels in the case $n = 7$.

B2. Basin of attraction of the cooperative stable fixed point

The measure $1 - p_k^{\mathrm{I}}(c)$ can be seen as a measure of the level of cooperation, as it measures the probability that cooperation evolves (as given by the size of the basin of attraction of the cooperative stable fixed point).

Result B2: if bistability exists for both k_1 and $k_1 + 1$, then $1 - p_{k_1+1}^{\mathrm{I}}(c) > 1 - p_{k_1}^{\mathrm{I}}(c)$.
Proof:
This follows directly from Property 2(iii), whereby $1 - p_k^{\mathrm{I}}(c)$ is the mirror image of $p_k^{\mathrm{II}}(c)$.
QED

For the example $n = 7$, Figure B2 represents $1 - p_k^{\mathrm{I}}(c)$ as a function of k, which illustrates that this measure is the mirror image of the measure under B1.

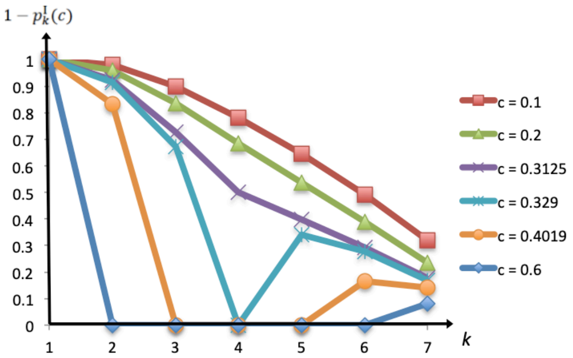

Figure B2. Effect of the threshold on the basin of attraction of the cooperative stable fixed point for several participation cost levels in the case $n = 7$.

B3. Ex-ante expected fraction of cooperating players

We next look at the ex-ante expected fraction of cooperating players, which includes the probability that cooperation evolves at all; this is the multiplication of the measures under B1 and B2, or $[1 - p_1^{\mathrm{I}}(c)]p_k^{\mathrm{II}}(c)$.

Result B3: effect of the threshold k on $[1 - p_1^{\mathrm{I}}(c)]p_k^{\mathrm{II}}(c)$ (ex-ante expected fraction of cooperation players):
(i) in case of a non-zero effect, the effect starting from $k = x$ is always minus the effect starting from $k = n - x + 1$;
(ii) for sufficiently large n, for a range of c just below $\bar{c}_x = \bar{c}_{n-x+1}$ (with $x = 2,3, \dots, (n - 1)/2)$), a higher threshold has a negative effect from $k = x - 1$ to $k = x$, and a positive effect from $k = n - x + 1$ to $k = n - x + 2$;
(iii) for sufficiently small participation costs, the threshold has a negative effect on the measure for low thresholds, and a positive effect for high thresholds.
Proof:
(i)
This follows directly from Property 2(iii).

(ii)
For $c = \bar{c}_x$, it is the case that $p_x^{\text{II}}(\bar{c}_x) = \frac{x-1}{n-1}$ if $x < \frac{n}{2}$, and that $p_x^{\text{I}}(\bar{c}_x) = \frac{x-1}{n-1}$ if $x > \frac{n}{2}$. Also, $\pi_{x-1}(p) = \pi_x(p)$ iff $p = \frac{x-1}{n}$. For n sufficiently large, the difference between $\frac{x-1}{n-1}$ and $\frac{x-1}{n}$ is very small, and so is the difference between $p_x^{\text{II}}(\bar{c}_x)$ and $p_{x-1}^{\text{II}}(\bar{c}_x)$ if $x < \frac{n}{2}$, and between $p_x^{\text{I}}(\bar{c}_x)$ and $p_{x-1}^{\text{I}}(\bar{c}_x)$ if $x > \frac{n}{2}$. Given this fact, for $c \approx \bar{c}_x$, if $x < \frac{n}{2}$ the fraction of cooperators in the cooperative stable fixed point hardly changes, but by Result B2 the basin of attraction decreases, so that the measure decreases; for $c \approx \bar{c}_x$, if $x > \frac{n}{2}$ the basin of attraction hardly changes, but by Result B1 the fraction of cooperators in the cooperative stable fixed point increases, so that the measure increases.
(iii)
For sufficiently small participation costs, the change in the fixed points does not vanish (contrary to what is the case for c close to $\bar{c}_x$ or 0). Looking at the derivative with respect to k of $[1 - p_k^{\text{I}}(c)]p_k^{\text{II}}(c)$, this equals $[1 - p_k^{\text{I}}(c)]\frac{\Delta p_k^{\text{II}}(c)}{\Delta k} + p_k^{\text{II}}(c)\frac{\Delta[1-p_k^{\text{I}}(c)]}{\Delta k}$. It follows that for lower thresholds, when by Figure 3 in the body of the paper the basin of attraction is large and the fixed-point fraction of cooperation players small, the first term of the effect dominates, which is positive; for high thresholds the basin of attraction is small and the fixed-point fraction of cooperators is small, so that the second term of the effect dominates, which is negative.
QED

Result B3 suggests that the U-shaped effect of Result 2 in the body of the paper, is reversed for sufficiently small participation costs, and is hill-shaped instead of U-shaped. This is confirmed by the example $n = 7$ in Figure B3, where we note that in line with Result B3, participation costs close to the maximal pivot probabilities for the different threshold levels are considered. Intuitively, by fixing participation costs in Figure 3 in the body of the paper and looking for the interior fixed points, it is clear that the fact that maximal pivot probabilities are large for small and for large thresholds, has the most impact when participation costs are large.

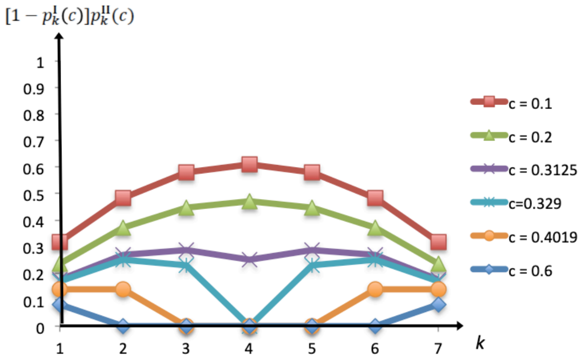

Figure B3. Effect of the threshold on ex-ante expected fraction of cooperating players for several participation cost levels in the case $n = 7$.

B4. Expected production of the public good in cooperative stable fixed point

The fact that in the cooperative stable fixed point, the fraction of cooperators is larger the larger threshold (see Section B1) is not that surprising as, if cooperation does evolve, more players are needed to achieve production of the public good. A more relevant measure for the level of cooperation may then simply be found in the expected production of the public good in the cooperative stable fixed point, denoted $Q_k(c)$, where $Q_k(c) = \sum_{\ell=k}^{n} \binom{n}{\ell} p_k^{\mathrm{II}}(c)^{\ell}[1 - p_k^{\mathrm{II}}(c)]^{n-\ell}$.

Result B4: effect of the threshold k on $Q_k(c) = \sum_{\ell=k}^{n} \binom{n}{\ell} p_k^{\mathrm{II}}(c)^{\ell}[1 - p_k^{\mathrm{II}}(c)]^{n-\ell}$ (expected production of the public good in cooperative stable fixed point):
(i) for any $k < n$, it is the case that $Q_n(c) > Q_k(c)$.
(ii) for sufficiently large n, for a range of c just below $\bar{c}_x$ (with $x = 2,3,\ldots,(n-1)/2$)), a higher threshold has a negative effect from $k = x - 1$ to $k = x$.
Proof:
(i)
For $k = n$, by Result 1(iii) in the body of the paper, it is the case that $p_n^{\mathrm{II}} = 1$, and the public good is produced with certainty. As for any smaller threshold $p_k^{\mathrm{II}} < 1$, the public good is not produced with certainty, and its expected value is smaller.
(ii)
In the same way as in the proof of Result B3(ii), we use the fact that the change in $p_k^{\mathrm{II}}(c)$ is small. Yet, the production of the public good decreases, as the threshold increases.
QED

Given that for small participation costs, the fraction of cooperating players increases monotonically over the full range of thresholds (see Result B1), Result B4 suggests that for sufficiently small participation costs, expected production of the public good in the cooperative stable fixed point increases in the threshold. This is confirmed for the example $n = 7$ in Figure B4. At the same time Figure B4 suggests that for all but the smallest levels of participation costs, expected production is a U-shaped function of the threshold.

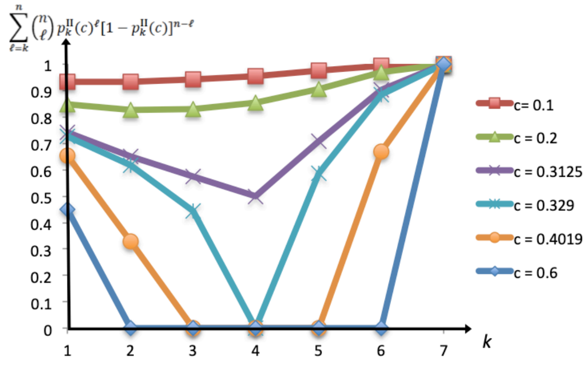

Figure B4. Effect of the threshold on the expected production of the public good in the cooperative stable fixed point for several participation cost levels in the case $n = 7$.

B5. Expected payoff in cooperative stable fixed point

While for $k = n$, in the cooperative stable fixed point the public good is produced with probability 1, expected participation costs of the players are also at a maximum. For this reason, one may instead consider as a measure of the level of (effective) cooperation at a cooperative stable fixed point, the expected payoff of the individual player, or the expected production of the public good minus the expected participation costs, which equals $U_k(c) = Q_k(c) - p_k^{\mathrm{II}}(c)c$.

Result B5: effect of the threshold k on $U_k(c) = Q_k(c) - p_k^{\mathrm{II}}(c)c$ (expected payoff in cooperative stable fixed point): it is the case that $U_1(c) = U_n(c) = 1 - c$, and that for $k \neq 1, n$, $U_k(c) < 1 - c$.
Proof:
For $k = 1$, as at the cooperative stable fixed point it is the case that $p_k^{\mathrm{II}}(c) = 1$, the public good is always produced, and the individual player always incurs participation costs c, so that the expected payoff is indeed $1 - c$. For all other k, at the interior cooperative stable fixed point, the individual player has the same expected payoff when she cooperates or defects, and may thus be expressed as the expected payoff she obtains when she cooperates, or $\sum_{\ell=k-1}^{n-1} \binom{n-1}{\ell} p_k^{\mathrm{II}}(c)^{\ell} [1 - p_k^{\mathrm{II}}(c)]^{n-1-\ell} - c$; this says that the expected payoff of a cooperator is given by the probability that $(k-1)$ or more of the $(n-1)$ other players in the group cooperate. Note now that for $k = 1$, the first term in this expression equals 1, showing that indeed $U_1(c) = 1 - c$. Since for any k such that $1 < k < n$, it is the case that the first term is smaller than 1, it follows that for $k \neq 1, n$, $U_k(c) < 1 - c$.
QED

Result B5 suggests that the expected payoff is a U-shaped function not only for large participation costs, but for all participation costs. This is confirmed by the example for $n = 7$ in Figure B5.

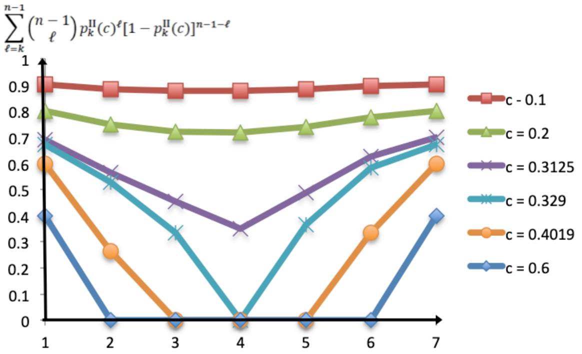

Figure B5. Effect of the threshold on the expected payoff in the cooperative stable fixed point for several participation cost levels in the case $n = 7$.

B6. Ex-ante expected production of the public good

While for $k = n$, in the cooperative stable fixed point the public good is produced with probability 1, at the same time by Result B2 the basin of attraction of this fixed point is at its minimum. To take into account the basin of attraction, we consider the ex-ante expected production of the public good, measured as $[1 - p_k^{\mathrm{I}}(c)]Q_k(c)$.

Result B6: effect of the threshold k on $[1 - p_k^{\mathrm{I}}(c)]Q_k(c)$ (ex-ante expected production of the public good in cooperative stable fixed point):
(i) $[1 - p_n^{\mathrm{I}}(c)]Q_n(c) < [1 - p_1^{\mathrm{I}}(c)]Q_1(c)$;
(ii) for sufficiently large n, for a range of c just below $\bar{c}_x$ (with $x = 2,3, \dots, (n-1)/2$)), a higher threshold has a negative effect from $k = x - 1$ to $k = x$;
(iii) for a range of c just below $\bar{c}_{n-x+1}$ (with $x = 2,3, \dots, (n-1)/2$), a higher threshold from $k = n - x + 1$ to $k = n - x + 2$ has the same effect as it has on Q.

Proof:
(i) This measure can be calculated to equal $1 - c^{n/(n-1)}$ for $k = 1$ and $1 - c^{1/(n-1)}$ for $k = n$, where the latter is lower.
(ii)
In the same way as in the proof of Result B3(ii), $p_k^{\mathrm{II}}(c)$ does not change; because the threshold increases, the expected production of the public good decreases, and by Result B2 additionally the basin of attraction of the cooperative stable fixed point decreases.
(iii)
In the same way as in the proof of Result B3(ii), the basin of attraction $[1 - p_k^{\mathrm{I}}(c)]$ does not change. It follows that the effect on $[1 - p_k^{\mathrm{I}}(c)]Q_k(c)$ is the same as the effect on $Q_k(c)$.
QED

Results B6(i) and B6(ii) suggest that, while the production of the public good in the cooperative stable fixed point may increase, the basin of attraction decreases sufficiently to make the ex-ante expected production of the public good decrease. Yet, as for larger thresholds and participation costs around the maximal pivot probability, the basin of attraction changes very little, the ex-ante expected production can still increase as well. This is confirmed by the example in Figure B6.

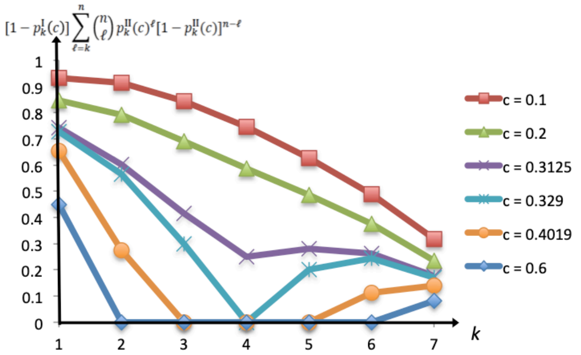

Figure B6. Effect of the threshold on the ex-ante expected production of the public good for several participation cost levels in the case $n = 7$.

B7. Ex-ante expected payoff

We finally look at the ex-ante expected payoff $[1 - p_k^{\mathrm{I}}(c)]U_k(c)$.

Result B7: effect of the threshold k on $[1 - p_k^{\mathrm{I}}(c)]U_k(c)$ (ex-ante expected production of the public good in cooperative stable fixed point):
(i) $[1 - p_n^{\mathrm{I}}(c)]U_n(c) < [1 - p_1^{\mathrm{I}}(c)]U_1(c)$;
(ii) for sufficiently large n, for a range of c just below $\bar{c}_x$ (with $x = 2,3, \dots, (n-1)/2$)), a higher threshold has a negative effect from $k = x - 1$ to $k = x$;
(iii) for a range of c just below $\bar{c}_{n-x+1}$ (with $x = 2,3, \dots, (n-1)/2$), a higher threshold from $k = n - x + 1$ to $k = n - x + 2$ has the same effect as it has on Q.
Proof:
(i) By Results B5(i), $U_n(c) = U_1(c)$, but by Result B2, the basin of attraction of the cooperative stable fixed point decreases in the threshold.
(ii) identical to Result B6(ii), where the result is reinforced because expected costs increase.
(iii) proof is identical to the one of Result B6(iii).
QED

Result B7(i) suggests that, while by Result B5 the expected payoff in the cooperative stable fixed point is a U-shaped function of the threshold, the decrease in the basin of attraction obtained in Result B2 is strong enough to make the ex-ante expected payoff decrease. Following Result B7(iii), as is confirmed in the example in Figure B7, an exception is found in large thresholds, where for participation costs close to the maximal pivot probabilities, ex-ante expected payoff locally increases, leading to a non-monotonic effect of the threshold.

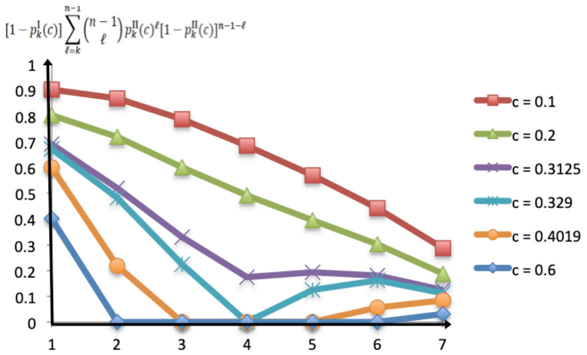

Figure B7. Effect of the threshold on the ex-ante expected payoff for several participation cost levels in the case $n = 7$.
